# Alteration of the Expression and Functional Activities of Myosin II Isoforms in Enlarged Hyperplastic Prostates

**DOI:** 10.3390/jpm14040381

**Published:** 2024-04-01

**Authors:** Xiao Wang, Weixiang He, Hui Chen, Rui Yang, Hongmei Su, Michael E. DiSanto, Xinhua Zhang

**Affiliations:** 1 Department of Urology, Renmin Hospital of Wuhan University, Wuhan 430071, China; wangxiao@whu.edu.cn (X.W.);; 2 Department of Urology, Xijing Hospital of the Fourth Military Medical University, Xi’an 710000, China; 3 Department of Surgery and Biomedical Sciences, Cooper Medical School of Rowan University, Camden, NJ 08103, USA; 4 Department of Urology, Zhongnan Hospital of Wuhan University, Wuhan 430071, China

**Keywords:** benign prostatic hyperplasia, lower urinary tract symptom, smooth muscle, myosin, organ bath

## Abstract

Introduction: Benign prostatic hyperplasia (BPH) is a common pathologic process in aging men, and the contraction of the prostatic smooth muscles (SMs) in the stroma plays a vital role in this pathogenesis, leading to lower urinary tract symptoms (LUTSs). The isoforms of both the SM myosin (SMM) and non-muscle myosin (NMM) are associated with the contraction type of the prostatic SMs, but the mechanism has not been fully elucidated. Methods: We collected prostate tissues from 30 BPH patients receiving surgical treatments, and normal human prostate samples were obtained from 12 brain-dead men. A testosterone-induced (T-induced) rat model was built, and the epithelial hyperplastic prostates were harvested. Competitive RT-PCR was used to detect the expression of SMM isoforms. We investigated the contractility of human prostate strips in vitro in an organ bath. Results: The results regarding the comparisons of SMM isoforms varied between rat models and human samples. In comparison with T-induced rats and controls, competitive RT-PCR failed to show any statistically significant difference regarding the compositions of SMM isoforms. For human prostates samples, BPH patients expressed more SM-1 isoforms (66.8% vs. 60.0%, *p* < 0.001) and myosin light chain-17b (MLC_17b_) (35.9% vs. 28.5%, *p* < 0.05) when compared to young donors. There was a significant decrease in prostate myosin heavy chain (MHC) expression in BPH patients, with a 66.4% decrease in MHC at the mRNA level and a 51.2% decrease at the protein level. The upregulated expression of non-muscle myosin heavy chain-B (NMMHC-B) was 1.6-fold at the mRNA level and 2.1-fold at the protein level. The organ bath study showed that isolated prostate strips from BPH patients produced slower tonic contraction compared to normal humans. Conclusion: In this study, we claim that in the enlarged prostates of patients undergoing surgeries, MHC expression significantly decreased compared to normal tissues, with elevated levels of SM-1, MLC_17b_, and NMMHC-B isoforms. Modifications in SMM and NMM might play a role in the tonic contractile properties of prostatic SMs and the development of LUTS/BPH. Understanding this mechanism might provide insights into the origins of LUTS/BPH and facilitate the identification of novel therapeutic targets.

## 1. Introduction

Benign prostatic hyperplasia (BPH) is a common pathologic process in aging men that is characterized by an increased number of epithelial and stromal cells in the periurethral and transition areas of the prostate [1,2]. An enlarged prostate can obstruct the bladder outlet, causing lower urinary tract symptoms (LUTSs). Meanwhile, the contraction of the smooth muscles (SMs) in the stroma results in a dynamic increase in prostatic urethral resistance, contributing up to about 50% of the total urethral pressure. This dynamic tone of the prostatic SMs is regulated mainly by the α_1_-adrenoreceptor [3,4]. Generally, the stimulation of the adrenergic nervous system leads to an increase in intracellular Ca^2+^ concentration [5]. Ca^2+^ binds with calmodulin and activates myosin light chain kinase, which then phosphorylates the myosin regulatory light chain (MLC_20_). This phosphorylation process induces the thin filaments to slide past the thick filaments in the SM cells, thus producing a contractile force [6].

In SM, the thick filaments are mainly composed of myosin, which is the molecular motor of the SM contractile apparatus. SM myosin (SMM) is a type II myosin composed of a pair of myosin heavy chains (MHCs) and two pairs of myosin light chains (MLCs) [6]. Studies have demonstrated that both the 3′ and 5′ ends of the MHC pre-mRNA are alternatively spliced to generate a COOH-terminal (SM-1 and SM-2 isoforms) and an NH_2_-terminal (SM-A and SM-B isoforms), respectively [7,8]. Moreover, the essential light chain MLC_17_ is alternatively spliced and has two 3′ end isoforms known as MLC_17a_ and MLC_17b_ [9,10]. The SMM isoform composition is known to affect the development and maintenance of force. In short, the SM-1, SM-A, and MLC_17b_ isoforms are associated with tonic-type contractions (such as in the aorta), characterized by a slow shortening speed (*V*_max_) but high force maintenance. On the other hand, the SM-2, SM-B, and MLC_17a_ isoforms are associated with phasic force generation (such as in the bladder), characterized by low force maintenance but fast *V*_max_ and rapid rates of force activation and relaxation [8,11,12]. The SM cells also express type II nonmuscle myosin (NMM II). Similar to SMM II, NMM II molecules are composed of three pairs of peptides: two MHCs, two regulatory light chains, and two essential light chains. There are at least three NM MHC isoforms (NMMHC-A, NMMHC-B, and NMMHC-C) in mammalian cells encoded by three different genes [13,14]. The NMMHC-A and NMMHC-B isoforms are similar in size to the SM MHC isoform, and the NMMHC-C isoform has 20 additional amino acids at the NH_2_ terminus. Though NMM is proposed as a housekeeping gene, it has reportedly been involved in the maintenance of SM tension [15]. Furthermore, the expression of NMM can be changed in some pathologic processes.

**Figure 1:**
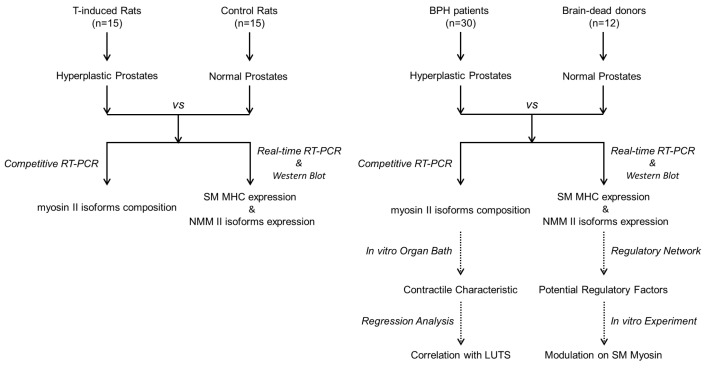
Schematic infographic of the study design.

Previously, we collected human hyperplastic prostate tissues from 10 patients undergoing radical cystectomy and found that hyperplasia prostate SM expressed more MHC, NMMHC-A, and NMMHC-B when compared to normal prostate tissues [16]. In this study, as shown in Figure 1, the inclusion criteria were extended, and we included 30 BPH patients who sought surgical treatment for moderate-to-severe LUTSs secondary to BPO or acute/chronic urine retention. We wished to give an updated evaluation of the expression of myosin II isoforms and the contractile properties of SM in the prostate tissues of clinical BPH patients. A testosterone-induced (T-induced) rat model was built, and we investigated the expression and activity of myosin II isoforms in hyperplastic prostates induced by testosterone. Moreover, we preliminarily investigated the potential regulatory factors in order to guide further studies.

## 2. Methods

### 2.1. Animal Protocol

The animal BPH model in this study involved Wistar rats induced with androgen. As we previously reported [17], a total of 30 male Wistar rats that were 12 weeks of age were divided into two groups: the BPH group (15 rats) and the control group (15 rats). The rats in the BPH group were subcutaneously injected with 2 mg testosterone propionate daily, whereas the control rats were similarly injected with an equivalent amount of sesame oil. Both groups were fed for 28 days. On day 29, all rats from both groups were weighed and then sacrificed under anesthesia. Blood was collected from the abdominal aorta to assess the serum testosterone level, which was performed via enzyme-linked immunosorbent assay according to the BlueGene Biotech (Shanghai, China) protocol. Whole ventral prostatic lobes, seminal vesicles, and the bladder were harvested and weighed. The surrounding prostatic capsules were dissected freely. The prostate tissues were fixed in 10% [*v*/*v*] neutral buffered formalin for 24–36 h and processed (routinely) for paraffin embedding. The paraffin-embedded tissue sections (4 μM) were stained with hematoxylin and eosin using standard techniques to determine the pathological changes in the prostate. Prostatic strips of approximately 1 × 1 × 0.5 cm were prepared for in vitro organ bath physiological studies and immediately placed in Krebs–Henseleit (Krebs) solution. Some of the remaining prostatic tissue was put into the RNA Sample Protector (Takara Bio. Inc., Otsu, Shiga, Japan) for polymerase chain reaction (PCR) analysis, and another portion was frozen in liquid nitrogen and preserved at −80 °C. 

### 2.2. Human Specimens

The human hyperplasic prostate specimens were derived from two sources, with a total of 30 patients who received surgical treatments for moderate-to-severe LUTSs secondary to BPO or acute/chronic urine retention. The prostatic tissues used for in vitro organ bath tests were sampled from five patients undergoing open enucleation. The samples were prepared as 1 × 1 × 0.5 cm strips and placed in Krebs solution. The other specimens were derived from 25 clinical BPH patients with enlarged prostates undergoing transurethral resection/enucleation of the prostate (TURP/TUEP). The following pre-surgical information from all 30 patients was recorded: demographics, PV calculated from an ultrasound image, pathology (epithelial hyperplasia, stromal hyperplasia, or equal proportions of epithelial and stromal hyperplasia), acute urine retention (AUR), international prostate symptom score (IPSS), postvoid residual urine volume (PVR), and maximum flow rate (Q_max_). Immediately after sampling, tissue specimens were frozen in liquid nitrogen and stored at −80 °C for subsequent analysis. Normal human prostate samples were obtained from 12 brain-dead men with a mean age of 29.1 ± 1.7 years undergoing organ donation surgery. 

### 2.3. Total RNA Extraction and Real-Time RT-PCR

Total RNA was isolated from the frozen tissues via the TaKaRa MiniBEST Universal RNA Extraction Kit (Takara Bio. Inc., Otsu, Shiga, Japan) according to the manufacturer’s protocol. Then, 100 ng of RNA was added to the one-step real-time RT-PCR reaction system (Takara Bio. Inc., Otsu, Shiga, Japan). The whole system was amplified in a 96-well plate at a reaction volume of 25 μL, with all samples run in triplicate using a CFX96 Touch Real-Time PCR Detection System (BioRad, Shanghai, China). Reverse transcription (42 °C for 5 min; 95 °C for 10 s) was first utilized, followed by an amplification program repeated for 40 cycles (95 °C for 5 s and then 60 °C for 30 s) using the SYBR Green measurement. For rat and human prostate tissues, the following targets were amplified: MHC, NMMHC-A, NMMHC-B, and NMMHC-C. The primer sequences are shown in Table 1. For relative quantification, gene expression was normalized to the expression of the GAPDH housekeeping gene and compared using the 2^−ΔΔCT^ method. 

### 2.4. Competitive RT-PCR

As previously reported [12,16,18], the differences in nucleic acid sequence in each pair of SMM isoforms were quite small. Because of this, competitive RT-PCR was used to detect the expression of SMM isoforms. PCR was performed using a total of 20 μL reaction volumes, including 2 μL of the RT product cDNA (50 ng/μL), 6 μL ddH_2_O, 1 μL each of upstream and downstream primer (50 μM), and 10 μL blue TSINGKE Master Mix (TSE004, TSINGKE, Wuhan, China). The alternatively spliced isoforms (SM-A/SM-B, SM-1/SM-2, and MLC_17a_/MLC_17b_) were amplified with competitive PCR using a GeneAmp 9700 thermal cycler (Applied Biosystems, Foster City, CA, USA). The primer sequences are shown in Table 1. The cycling conditions started at 94 °C for 5 min, followed by 35 cycles (30 s at 94 °C, 30 s at 55 °C, and 120 s at 72 °C), then ending with a one-time 7 min incubation at 72 °C to ensure the extension of all products. Because all three pairs of myosin isoforms were generated via alternative splicing, there was an opportunity to perform competitive PCR for each primer pair by designing primers that flanked the insert region.

The PCR products were then separated via electrophoresis on a 2.5% agarose gel and visualized using GelStar staining and ultraviolet illumination. Band density was quantified by reflectance scanning of gel photographs obtained with a BioDoc-It camera set-up (UVP; Upland, CA, USA) using a Bio-Rad (Hercules, CA, USA) GS-700 imaging densitometer, and subsequent analyses used the Bio-Rad Molecular Analyst 1D program that enabled us to obtain quantitative relative SMM isoform expression data for all isoform pairs. Sequencing analysis with the PCR product clones was conducted to discriminate the nonspecific bands.

### 2.5. In Vitro Organ Bath Studies

As previously described [12,16,18], rat and human prostate strips were mounted longitudinally in a 4 mL organ bath, Multi Myograph Model 810MS (Danish Myo Technology; Aarhus, Denmark). The myograph was connected in line to a PowerLab 4/30 Data Acquisition System (ADInstruments; Colorado Springs, CO, USA) and then to a Pentium Dual-Core processor computer, which allowed us to monitor the physiological force in real time. The SM strips were equilibrated for at least 1 h in Krebs buffer at 37 °C with continuous bubbling of 95% O_2_ and 5% CO_2_. The buffer had the following mM composition: NaCl, 110; KCl, 4.8; CaCl_2_, 2.5; MgSO_4_, 1.2; KH_2_PO_4_, 1.2; NaHCO_3_, 25; and dextrose, 11, with replacement every 15 min. The strips were continuously adjusted to resting tension. After equilibration, the tissues were contracted using 60 mM KCl. This degree of contractile response was considered 100%, and the force induced by different concentrations of phenylephrine (PE) was expressed as a percentage of this value.

### 2.6. SDS-PAGE and Western Blot Analysis

Proteins were extracted from frozen samples using the CelLytic™ NuCLEAR™ Extraction Kit (Sigma-Aldrich, Beijing, China), and then, 100 μg of each sample was electrophoresed on a 10% sodium dodecyl sulfate-polyacrylamide (SDS-PAGE) gel (Wuhan Boster Biological Technology, Ltd., Wuhan, China) and transferred to a polyvinylidene fluoride (PVDF) membrane (Millipore, Billerica, MA, USA) using a Bio-Rad wet transfer system. The membrane was blocked for 2 h at room temperature with Tris-buffered saline with 0.1% [*v*/*v*] Tween (TBST) containing 5% [*w*/*v*] nonfat dry milk solution. The membrane was incubated overnight with the primary antibody (shown in Table 2). Membranes were washed with TBST thrice and then incubated with the secondary antibody at room temperature for 2 h. The reaction antigen was detected with an enhanced chemiluminescence (ECL) kit (Thermo Scientific Fisher, Waltham, MA, USA). The bands were quantified by reflectance scanning of gel photographs obtained with a BioDoc XRS+ camera using Bio-Rad Molecular Imager^®^ ChemiDoc™ XRS+ System and Quantity One^®^ SW 1-D Analysis Software version 4.6 (Bio-Rad).

### 2.7. Immunofluorescence

Human prostate tissues were embedded in Tissue-Tec OCT compound (Sakura Finetek Japan, Tokyo, Japan) and snap frozen. The tissues were then sectioned into 10 μM slices and then thawed, mounted onto glass slides using a cryostat (Leica CM 1850, Wetzlar, Germany), air-dried, and fixed for 10 min in ice-cold acetone. Slides were washed in phosphate-buffered saline (PBS) and then incubated for 2 h in a mixture of PBS supplemented with 0.2% [*v*/*v*] Triton X-100 and 0.1% [*w*/*v*] bovine serum albumin, followed by overnight incubation with the primary antibody (Table 2). The secondary antibodies employed to visualize the localization of the primary antibodies (Jackson ImmunoResearch Inc., West Grove, PA, USA) were Cy3-conjugated goat antirabbit IgG (1:1000) and Cy2-conjugated donkey antigoat IgG (1:400), and DAPI was used to stain the nucleus. Colocalization analysis was performed using NIS-Elements Viewer 3.20 (Nikon, Japan). Visualization was performed with a laser microscope (Olympus, Tokyo, Japan).

### 2.8. Regulatory Network Analysis

The array dataset GSE119195 deposited by our team was used for subsequent analysis [19]. The raw data were preprocessed via the robust multiarray average algorithm with the use of oligo in the R software of Bioconductor version 4.2.3 (Seattle, WA, USA). The differentially expressed genes (DEGs) in the hyperplastic prostate samples compared to normal prostate samples were analyzed using the limma package in Bioconductor. The *p*-values of the DEGs were calculated using the unpaired Student’s *t*-test in the limma package. A fold change of ≥2 and *p* < 0.05 were set as the cut-off criteria. The differentially expressed microRNAs (DEMs) were derived from Zhang’s previous study of microRNA expression profiles in BPH [18]. The TRANSFAC database was used to find transcription factors (TFs) targeting MYH11 and MYH10 among the DEGs, and DIANA TOOLS was used to predict the DEMs that targeted MYH11, MYH10, and relative TFs. Subsequently, the cytoscape plugin iRegulon was used to analyze TFs regulating coexpressed genes and microRNAs [20]. Finally, regulatory networks were constructed by merging selected TFs-DEGs and DEMs-DEGs pairs using Cytoscape. Considering that SMM plays a vital role in the contractile dysfunction of smooth muscles in the enlarged prostate, we selected miR-106a-5p, KLF6, and NR4A2 to further assess their effects on the expression of MYH11.

### 2.9. Cell Culture, Transfection, and Immunofluorescence

SV40 large-T antigen-immortalized stromal cell line WPMY-1 (Cat. #GNHu36) was purchased from the Stem Cell Bank of the Chinese Academy of Sciences in Shanghai, China. WPMY-1 cells were cultured in DMEM medium (Gibco, Shanghai, China) containing 1% penicillin G sodium/streptomycin sulfate, and 5% FBS in a humidified atmosphere consisting of 95% air and 5% CO_2_ at 37 °C. The miR-106a-5p mimic, miR-106a-5p inhibitor, mimics control, and inhibitor control were all purchased from GenePharma (Shanghai, China). Two small interfering RNA (siRNA) oligonucleotides for human KLF6 and NR4A2 were synthesized from GenePharma (Shanghai, China). Overexpression was achieved by inserting PCR-amplified coding sequences of human KLF6 and NR4A2 into the pIRES2-ZsGreen1 expression vector (Clontech, Shanghai, China). Cell transfection was performed with the Lipofectamine 2000 transfection reagent (Invitrogen) according to the manufacturer’s recommendation. For cell immunofluorescence microscopy, cells were seeded on 12 mm coverslips and washed using ice-cold PBS, pH = 7.4). The coverslips were then fixed with 4% paraformaldehyde (PFA) for 30 min, treated in 0.1% Triton X-100, and then blocked in goat serum for 30 min at room temperature. The KLF6, NR4A2, and MHC primary antibodies (Table 2) were used for incubation. DAPI was used for staining the nucleus. Visualization was performed using a laser scanning confocal microscope (Olympus, Tokyo, Japan).

### 2.10. Statistical Analysis

The data were processed using SPSS 17.0 for Windows. After performing the normality test and variance equality test, Student’s *t*-tests were used for the statistical analysis of the continuous variable means. Linear regression and logistic regression were employed to evaluate the effect of the influencing factors on PVR, AUR, Qmax, and IPSS. The probability of a type I error was considered at 0.05, and all tests were two-sided.

### 2.11. Ethical Standards

All procedures involving human participants were performed in accordance with the ethical standards of the research committee of Wuhan University and the principles of the Declaration of Helsinki. All participants gave written informed consent before participating in the study. All procedures involving animals were performed in accordance with the standard care and use of laboratory animals (National Research Council, Washington, DC, USA) and the ethical standards of Wuhan University. All experimental protocols were approved by the research committee of Wuhan University.

## 3. Results

**Figure 2:**
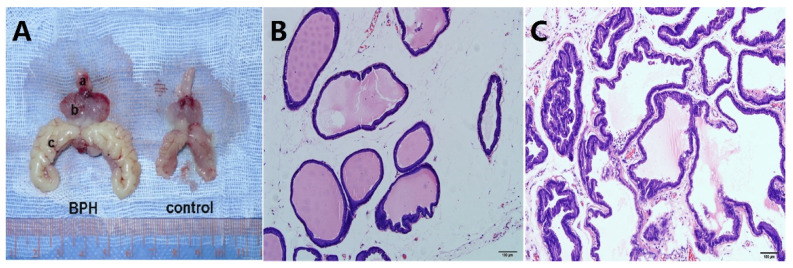
Typical photograph and histological examination of the prostate from a BPH and control rat. (**A**) Typical photographs of a BPH and control rat. (a) Bladder, (b) ventral prostate, and (c) seminal vesicle. HE staining of prostate tissue for (**B**) normal rat prostate and (**C**) BPH rat prostate.

The T-supplementation rat model of BPH was validated through the 1.6- and 2-fold increases in the weight of the ventral prostate and seminal vesicle (Table 3), respectively. Figure 2A shows photographs of typical BPH and control rats. As expected, the BPH rats had significantly increased T levels (Table 3, *p* = 0.016), but they also had significantly decreased body weight (Table 3, *p* < 0.001). They also had a 1.8-fold increase in prostate index [prostate wet weight (mg)/body weight (g)] (Table 3, *p* < 0.001). The histological examination revealed hyperplasia of the prostate in the epithelium, specifically in the thickened epithelial layer, papillary fronds protruding into the glandular cavities, and a decreased stromal component (Figure 2B,C).

**Figure 3:**
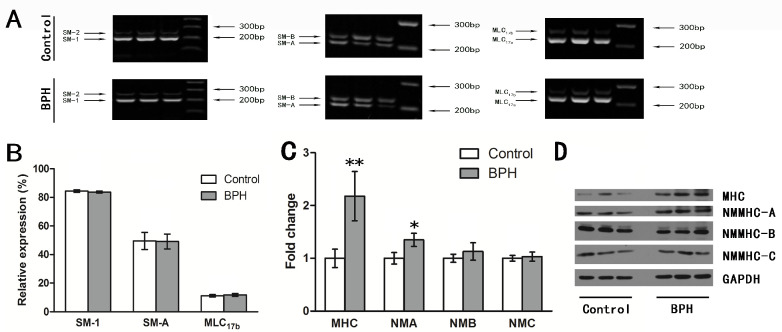
Alteration to the expression of myosin II for BPH and control rats. (**A**) Typical competitive RT-PCR bands of SMM II isoforms for prostate tissues from BPH and control rats. (**B**) The bar graph shows the relative expressions of SM-1, SM-A, and MLC_17b_ between different groups (n = 15 different rats for each group). (**C**) Relative mRNA expression of the target genes evaluated using real-time RT-PCR in rat prostate (n = 15 different rats for each group). (**D**) Representative Western blotting bands and summary graphs of target proteins and GAPDH in the rat prostate. Quantification of protein expression was calculated using the gray value ration of the target protein/GAPDH. All values are shown as their mean ± SD. * *p* < 0.05 vs. control; ** *p* < 0.01 vs. control.

**Figure 4:**
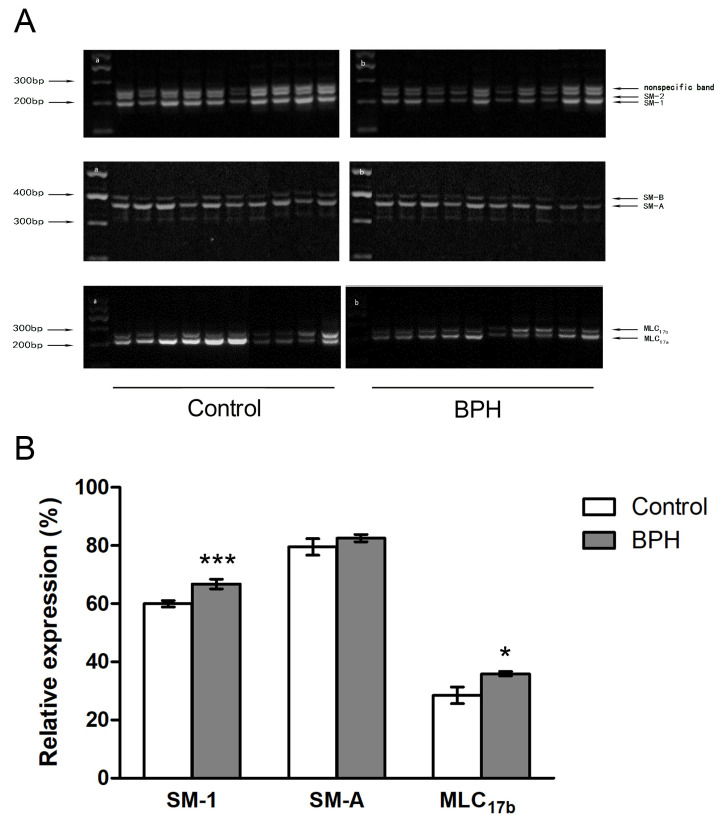
Composition of SMM II isoforms in prostates from normal humans and BPH patients. (**A**) Typical competitive RT-PCR bands of SMM II isoforms for prostate tissues from normal humans (n = 12) and BPH patients (n = 30). (**B**) The bar graph for the relative expressions of SM-1, SM-A, and MLC_17b_ in normal humans (n = 12) and BPH patients (n = 30). All values are shown as their mean ± SD. * *p* < 0.05 vs. control; *** *p* < 0.001 vs. control.

**Figure 5:**
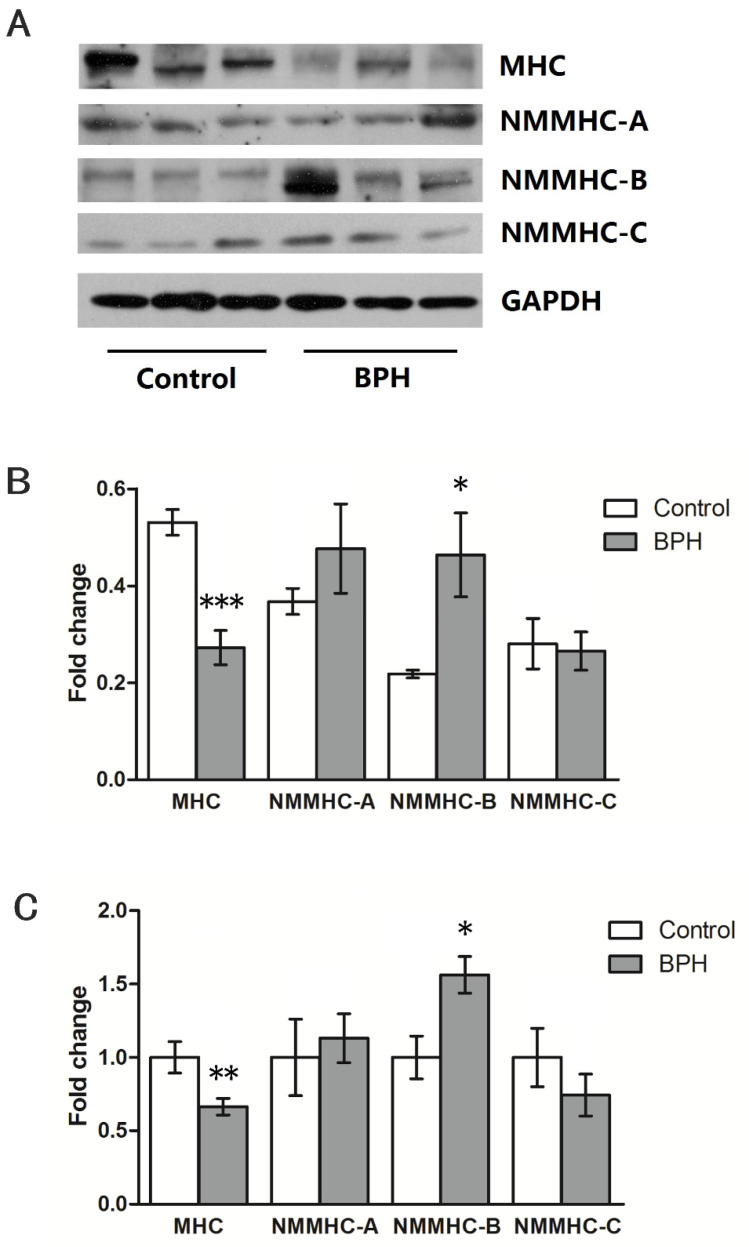
Expression of SM MHC and NMM II isoforms in human prostate. (**A**) Representative Western blotting bands of target proteins and GAPDH in the human prostate. (**B**) The summary graph of relative protein expression in the human prostate (n = 5 different humans for each group). Quantification of protein expression was calculated using the gray value ration of target protein/GAPDH. (**C**) Relative mRNA expression of target genes from normal humans (n = 12) and BPH patients (n = 30). All values are shown as their mean ± SD. Experiments were repeated three times for each sample. * *p* < 0.05 vs. control; ** *p* < 0.01 vs. control; *** *p* < 0.001 vs. control.

Several kinds of examinations were used to compare the myosin II isoforms of the rat prostates from the BPH model with the controls, as shown in Figure 3. Competitive RT-PCR showed that the rat prostate expressed 84.4% SM-1, 49.5% SM-A, and 11.1% MLC_17b_, but the comparison failed to show any statistically significant difference regarding the compositions of SMM isoforms (Figure 3A,B). The prostates of the BPH model rats upregulated MHC expression two-fold at the mRNA level and more than three-fold at the protein level (Figure 3C,D). Furthermore, as confirmed by real-time quantitative RT-PCR and Western blotting analysis, the prostates of the BPH rats had increased expression of NMMHC-A vs. normal rats (Figure 3C,D). For the human samples, normal prostate SM expressed almost 60.0% SM-1, more than 79.6% SM-A, and 28.5% MLC_17b_, as shown in Figure 4. In the prostate tissue of BPH patients, the relative expression of mRNA transcript in the SM-1 isoforms was 66.8%, which was significantly different compared to the controls. Meanwhile, the MLC_17b_ isoform was also upregulated significantly, with an expression of 35.9%. The composition of SM-A increased slightly in the hyperplastic gland compared to the normal prostate, though no statistical significance could be tested either. These alterations in SMM isoforms indicate that the contractile characteristic of hyperplastic prostatic smooth muscles switched to a more tonic contractile phenotype. Further examination focusing on SM MHC and NM MHC was performed using real-time RT-PCR and Western blotting analysis. As shown in Figure 5, there was a significant decrease in prostate MHC expression in BPH patients, and there was upregulated expression of NMMHC-B. We found a 66.4% decrease in MHC at the mRNA level and a 51.2% decrease at the protein level, and the expression of NMMHC-B increased 1.6-fold at the mRNA level and 2.1-fold at the protein level.

**Figure 6:**
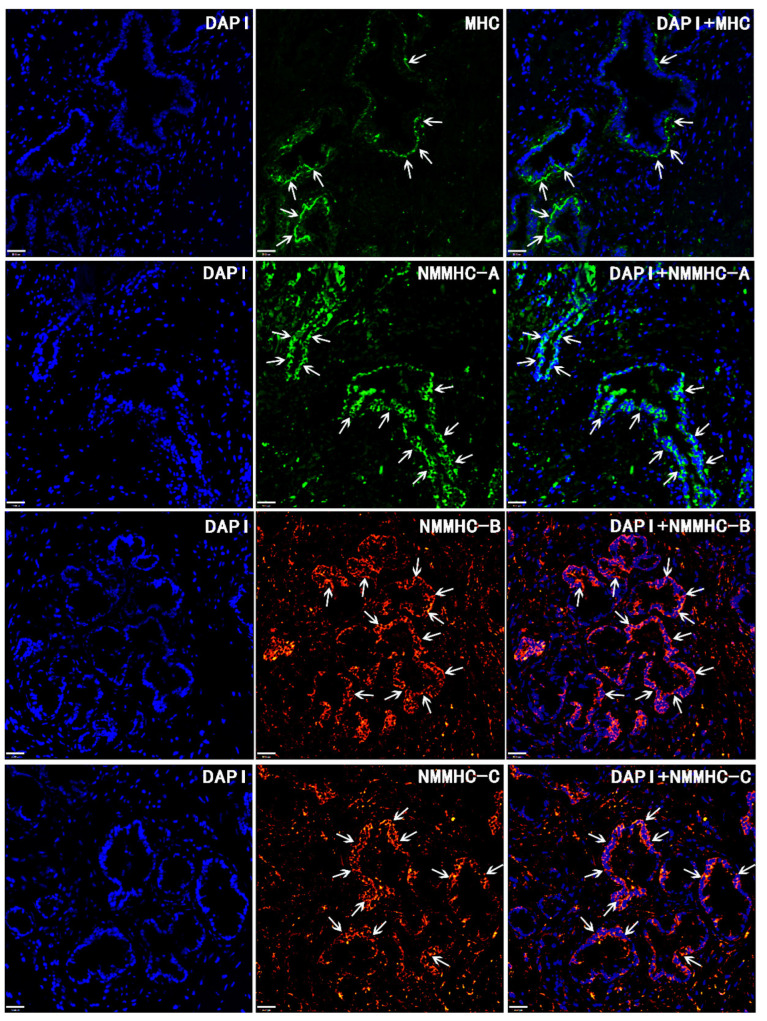
Immunolocalization of SM MHC and NM MHC isoforms in human enlarged prostate. SM MHC and NM MHC were distributed in different components of the enlarged prostate. NMM isoforms (NMMHC-A, B, and C) were more abundant in epithelial compartments (showed by white arrows), and SM MHC was located only in the basal layer of the hyperplastic prostatic stroma circling the glandule (showed by white arrows). Nuclei were stained using DAPI (blue). The images were photographed by confocal fluorescence microscopy (×200 magnification).

Immunofluorescence studies were conducted to accurately localize the expression of SM MHC and NM MHC in prostate tissues derived from BPH patients (Figure 6). Using a laser confocal microscope, we found that all NMM isoforms (NMMHC-A, B, and C) could be detected in both the stromal and epithelial compartments, with NMM expression being more abundant in epithelial components. However, in the enlarged prostate, SM MHC was rarely expressed in the stromal components, which was inconsistent with our previous study. SM MHC was detected only in the basal layer of the hyperplastic prostate stroma circling the glandule.

Table 4 shows the demographics and preoperative parameters of the 30 patients, and Table 5 shows the correlations between these parameters and the expression of SMM and NMM isoforms. As shown in Table 4, the mean age of the 30 patients was 71.2 (range: 51–84) years. All patients had enlarged prostates with PVs. ranging from 50.5 to 105.4 mL. There were 18 (60.0%) patients with postsurgical histologic epithelial hyperplasia, and 7 (23.3%) patients had equal proportions of epithelial and stromal hyperplasia of the prostate. Only five (16.7%) cases were stromal hyperplasia. Limited information can be extracted from Table 5, which details the investigation into the composition of SM-A in the prostatic SM, which was correlated with patients’ Qmax, and the percentage of SM-1 could affect the patients’ LUTSs, as evaluated by IPSS. The correlation was still statistically significant after adjusting for age, PV, pathology, and chronic diseases. The expression level of SM MHC was also an influencing factor for IPSS. Moreover, the expression of NMMHC-B was related to patients’ PVR and IPSS, but the significance could not be detected, even after adjusting for patient demographics.

**Figure 7:**
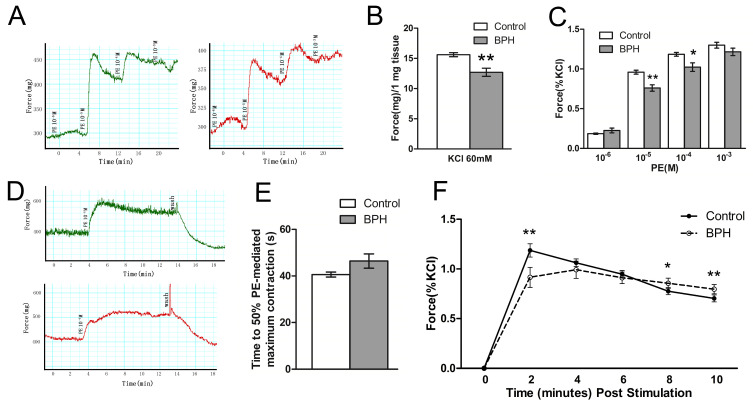
In vitro contractility of human prostate strips and analysis. (**A**) Force tracing curves of prostate strips in response to increasing doses (10^−6^ to 10^−3^ M) of PE from normal humans and BPH patients. (**B**) Summary graph for the in vitro contraction force of prostate strips in response to 60 mM KCl. (**C**) Summary graph for the in vitro contraction force of prostate strips in response to increasing doses (10^−6^ to 10^−3^ M) of PE. (**D**) Force tracing curves of prostate strips after stimulation with 10^−4^ M PE. (**E**) Summary graph of time to 50% of PE-induced maximum contraction for prostate strips. (**F**) Line graph of force maintenance of prostate strips after stimulation with 10^−4^ M PE. Prostate strips were obtained from five different humans for each group; one strip was used for each human. * *p* < 0.05 vs. control; ** *p* < 0.01 vs. control.

We investigated prostate contractility in vitro. As shown in Figure 7, prostatic SM generated a significant force in response to KCl depolarization and PE-mediated adrenergic stimulation in a dose-dependent manner. Figure 7A illustrates the representative tracing of human prostate SM contraction in response to the different concentrations of PE. These PE dose–response contractions were normalized to KCl-elicited force, and they are averaged in Figure 7B. Overall, the isolated prostate strips from BPH patients produced a slower tonic contraction compared to normal humans. The normal prostate strips produced an average tissue tension of 15.6 mg force/mg at 60 mM KCL, which was reduced significantly in BPH patients. Meanwhile, the contraction of the enlarged hyperplastic prostate strips decreased significantly in response to 10^−5^ and 10^−4^ M PE. The hyperplastic prostate strips needed more time to achieve 50% maximum contraction when mediated by PE, indicating a reduced velocity of contraction compared to the normal prostate, though no statistical difference could be tested (Figure 7E). In spite of this weakened contraction, force maintenance was enhanced for the prostate strips from BPH patients. The hyperplastic prostate strips reserved 85.5% and 79.6% force tension at 8 and 10 min after 10^−4^ M PE stimulation, respectively, and these were significantly higher than the 77.6% and 70.3% tension maintained by the normal prostate strips at the same time periods (Figure 7F).

**Figure 8:**
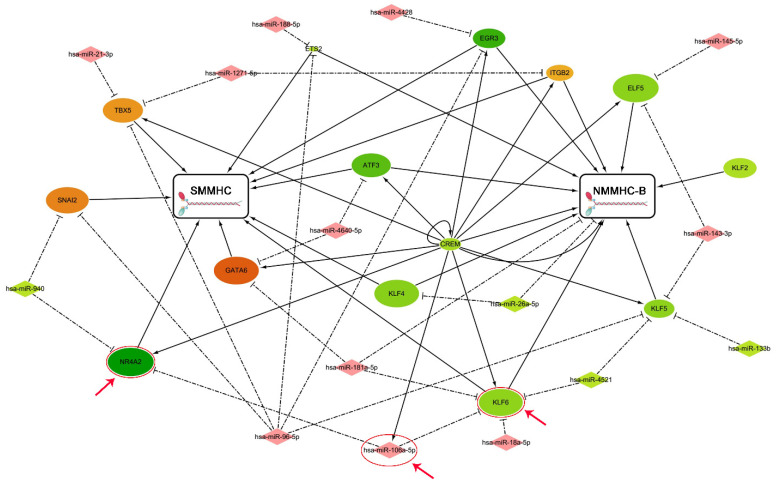
Regulatory networks of differently expressed transcription factors and microRNAs in enlarged hyperplastic prostate targeting SM MHC and NMMHC-B. Transcription factors are shown as ovals, and microRNAs are shown as diamonds. *p*-values determine the size of the ovals, and the different colors represent expression distribution. Red indicates high expression, and green indicates low expression. Finally, we analyzed miR-106a-5p and two TFs (KLF6 and NR4A2) as the red arrows showed, to subsequently validate the miR-106a-5p-KLF6-MYH11 and miR-106a-5p-NR4A2-MYH11 pathways in the prostate in vitro.

**Figure 9:**
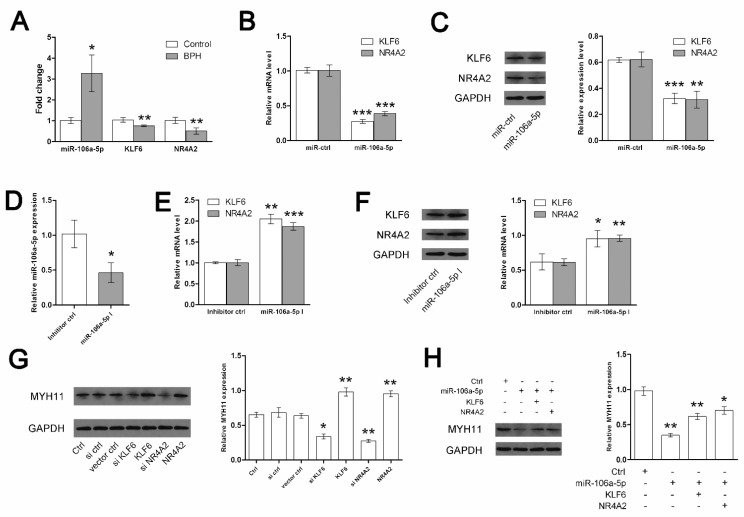
Validation of the miR-106a-5p-KLF6-MYH11 and miR-106a-5p-NR4A2-MYH11 pathways in WPMY−1 cell lines. (**A**) Relative mRNA expression of target genes from normal humans and BPH patients, determined by quantitative real-time PCR (n = 5 for each group). (**B**) Relative mRNA expression of KLF6 and NR4A2, determined by quantitative real-time PCR after interference of miR-106a-5p in the WPMY−1 cell lines. (**C**) Western blotting bands and a summary graph of the relative protein expression of KLF6 and NR4A2 after interference of miR-106a-5p in the WPMY−1 cell lines. (**D**) Verification of miR-106a-5p inhibition. (**E**) Relative mRNA expression of KLF6 and NR4A2, determined by quantitative real-time PCR after miR-106a-5p inhibition. (**F**) Western blotting bands and a summary graph of the relative protein expression of KLF6 and NR4A2 after miR-106a-5p inhibition. (**G**) Western blotting bands and a summary graph of the protein expression of MYH11 in the WPMY−1 cell lines after overexpression and interference of KLF6 and NR4A2, respectively. (**H**) Western blotting bands and a summary graph of the protein expression of MYH11 in the WPMY−1 cell lines after transfection of miR-106a-5p only and co-transfection with KLF6 and NR4A2. All values are shown as their mean ± SD. Experiments were repeated three times for each sample. * *p* < 0.05 vs. control; ** *p* < 0.01 vs. control; *** *p* < 0.001 vs. control.

**Figure 10:**
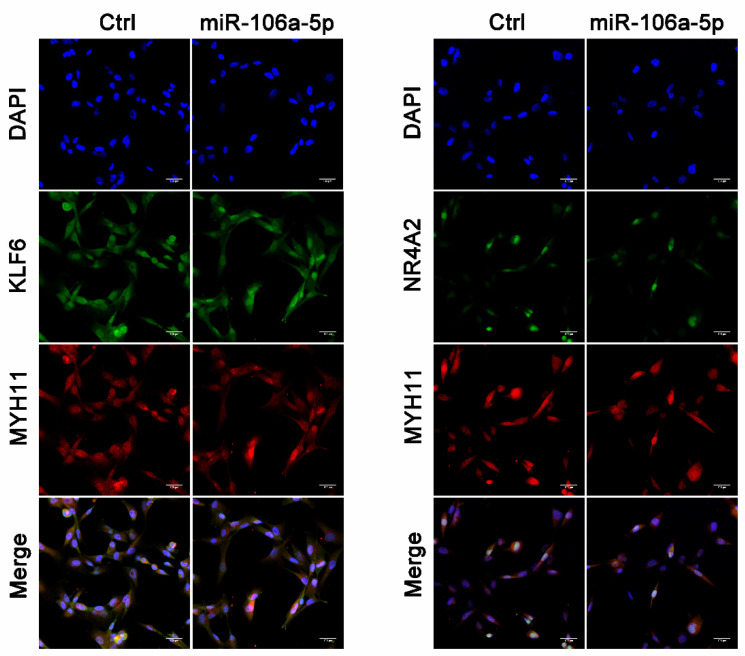
Immunolocalization of KLF6, NR4A2, and MYH11 in WPMY-1 cell lines and the effect of miR-106a-5p interference. DAPI (blue), transcription factors (green fluorescence), MYH11 (red fluorescence), and the merged images are shown from top to bottom (×400 magnification). Fluorescence intensity was downregulated after interference with miR-106a-5p.

To subsequently explore the regulatory factors of the smooth muscle myosin in the human prostate, we focused on the TFs and microRNAs targeting the MYH11 gene encoding SM MHC, as well as the MYH10 gene encoding NMMHC-B. Using the TRANSFAC database and DIANA TOOLS, we recognized the differently expressed transcription factors (DETFs) and microRNAs (DEMs) that were derived from gene expression profiling (GSE119195) and seen in a previous study from Zhang [18]. Finally, 14 DETFs and 15 DEMs were retrieved to build the regulatory networks targeting SM MHC and NMMHC-B (Figure 8). Then, considering that SMM plays a crucial role in the contractile dysfunction of the enlarged prostate, we used miR-106a-5p and two TFs (KLF6 and NR4A2) to validate the miR-106a-5p-KLF6-MYH11 and miR-106a-5p-NR4A2-MYH11 pathways. Quantitative real-time PCR determined that the expression of miR-106a-5p, KLF6, and NR4A2 differed in the prostate tissues of BPH patients and controls (Figure 9A). We found that the expression of miR-106a-5p could interfere with the expression of KLF6 and NR4A2 at both the mRNA (Figure 9B) and protein levels (Figure 9C). Thus, both KLF6 and NR4A2 could be upregulated by inhibiting miR-106a-5p in WPMY-1 cell lines (Figure 9E,F). At the protein level, the expression of MYH11 could be downregulated by interfering with KLF6 and NR4A2 and, conversely, be upregulated by overexpressing KLF6 and NR4A2 (Figure 9G). Meanwhile, the expression of MYH11 could also be disrupted by miR-106a-5p, but this interference could be rescued by transfecting KLF6 and NR4A2 (Figure 9H). As shown in Figure 10, in the study of human cell immunofluorescence, MHCs were stained green and found to be expressed in the nucleus and cytoplasm of the WPMY-1 cell. After transfection, miR-106a-5p, KLF6 (expressed in the nucleus and cytoplasm), NR4A2 (expressed mainly in the nucleus), and MYH11 (expressed in the nucleus and cytoplasm) were downregulated.

## 4. Discussion

In the present study, we collected human hyperplastic prostate tissues from BPH patients undergoing surgical treatments for moderate-to-severe LUTSs secondary to BPO or acute/chronic urine retention. All the patients met the surgical criteria, with prostate volumes enlarged by more than 50 mL. We found that in enlarged prostates, MHC expression significantly decreased, with increased levels of SM-1, MLC_17b_, and NMMHC-B isoforms. Recently, our team published a study in which we investigated the expression and activity of SMM and NMM in human hyperplastic prostates [16]. In our previous study, the hyperplastic prostate tissues were obtained from patients undergoing radical cystectomy for bladder cancer, as diagnosed by pathologists, but the prostate volumes were small, and the histological examination of most cases showed obvious stromal hyperplasia. Since the pathology of prostate hyperplasia varies depending on prostate volume, as the gland grows, epithelial elements become predominant, and most patients with enlarged glands have epithelial hyperplasia [21]. Moreover, a histologic study showed that prostate tissues from Chinese patients had higher glandular densities compared to samples from American patients [22]. Considering the histologic differences, in this study, we wished to further evaluate the expression of myosin II isoforms and the contractile properties of SM in enlarged prostates with epithelial hyperplasia. 

We aimed to build BPH rat models with epithelial hyperplasia to simulate the pathogenic process of BPH patients with enlarged prostates. Our T-induced BPH rat model was validated through the presence of hyperplasia and significantly increased prostate gland and seminal vesicle (androgen-sensitive organs) weights. We observed body weight loss with T supplementation, and this might be due to increased daily activity and an increased ratio of lean body mass to fat body mass. Similar to previous observations [17], T injection caused notable acinar epithelium hyperplasia; more specifically, an increased number of acini, papillary fronds protruding into the glandular cavities, and a thickening of the epithelial layer. Our previous study using Masson’s trichrome staining quantified that the BPH rats’ epithelia significantly increased compared to controls, even though the SM content remained unchanged [17]. We tried to explore the difference in SMM and NMM between BPH rats and controls, but positive results were rarely detected. More specifically, the SMM isoforms of the BPH model did not show any statistically significant difference when compared to controlled rats. However, in our previous study, T deprivation could alter the SMM isoform composition with the upregulation of SM-B and SM-2 and with the downregulation of MLC17a [12]. Moreover, the expression of MHC and NMMHC-A in the rat prostate was elevated with T supplementation, and this was verified at the mRNA and protein levels. In accordance with a study by Thiyagarajan et al., the contractile profiles of rat hyperplastic prostate strips increased in response to KCl depolarization and PE-mediated adrenergic stimulation in a dose-dependent manner compared to controls [23]. This elevated contractile function may be due to the increased sensitivity of the prostate to α-adrenoceptor agonists or the upregulated SM contractile apparatus. In our previous study [12], we found that both the supplementation and deprivation of T could upregulate the expression of MHC in the rat prostate. This may be because T plays different roles in these two processes. It is widely understood that androgen withdrawal causes the activation of specific genes involved in epithelial programmed cell death, but stromal cells were resistant to the effect of short-term androgen deprivation [24,25]. In our previous study, castration mainly led to a marked involution of the glandular compartment and correlated to a loss of epithelial cells, and the relative volume of the stroma increased (2.3-fold increase in SM and 2.1-fold increase in collagen fibers). The increased SM and relatively decreased epithelial cells contributed to a greater expression of MHC at the mRNA and protein levels. The reference used was GAPDH, which was expressed in both epithelial and SM cells. However, in our T-induced rat BPH model, T may activate the downstream androgen-dependent genes and relative growth factors [26], thus upregulating MHC expression despite the SM content remaining unchanged. In humans, this implies that androgen withdrawal can cause partial involution of established BPH [27], but androgen supplementation cannot cause BPH or serve as the direct mitogen for prostate growth in aging men [28]. Thus, despite the increased size of the rat prostates and T-induced histological alteration of the glandular compartment, this rat BPH model is not an absolute reflection of the pathological changes in human hyperplastic prostates.

The compositions of the SMM isoforms were associated with the contraction phenotypes. In summary, the SM-B, MLC_17a_, and SM-2 isoforms were associated with fast phasic contraction, and SM expressing more SM-A, MLC_17b_, and SM-1 were associated with slow tonic contraction. Compared with normal prostate tissues from young males, the compositions of SMM isoforms were altered in the hyperplastic prostates from BPH patients. The percentages of SM-1 and MLC_17b_ in the prostatic SM increased for BPH patients, while the differences in SM-A composition had no statistical significance compared to normal tissues. These alterations to SMM isoforms represent the contractile characteristic of enlarged prostate smooth muscles switching to a more tonic contractile phenotype, which was consistent with our previous study on the small hyperplastic prostate [16]. The most important difference with our previous study was that the expression of SM MHC decreased significantly in the enlarged prostate of BPH patients. This finding was more consistent with a study by Lin et al. [29], but the changes were more modest. We then performed immunofluorescence studies to accurately localize SM MHC expression in the prostate tissues of BPH patients with enlarged prostates. We found that SM MHC was detected only in the basal layer of the stroma but was rarely expressed in the stromal components. This could be because myosin was degraded in the SM cells of the enlarged prostate, maybe due to an impaired senescence mechanism, or because SM cells were only located in the basal layer circling the glandular components in the enlarged prostate with epithelial hyperplasia. We also found that the expression of NMMHC-B was elevated in the hyperplastic prostate compared to normal tissues, but the expression of NMMHC-A remained unchanged. We have investigated (in vitro) that NMMHC-B could modulate the proliferation and apoptosis of prostatic epithelial cells. In fact, NMMHC-B has been correlated with SM tonic contraction and may play an ancillary role in tension maintenance [30,31]. Confocal tests revealed that NMMHC-B expression was located both in glandular and stromal components, though this was more abundant in epithelial cells.

Further in vitro organ bath tests confirmed the alteration to the contractile profiles of prostate strips from BPH patients. Our study showed that force generation of hyperplastic prostate strips reduced significantly in response to KCl depolarization and high concentrations of PE (10^−5^ and 10^−4^ M). Meanwhile, the prostate strips from BPH patients took more time to achieve 50% of the PE-mediated maximum contraction, representing a slower shortening velocity compared to the normal prostate, though the difference was not statistically significant. Aside from the weakened force generation and slower shortening velocity, force maintenance was also enhanced for hyperplastic prostate strips. The prostate strips from BPH patients showed 85.5% and 79.6% force tension at 8 and 10 min after 10^−4^ M PE stimulation, respectively, and these results were significantly higher than normal prostate strips. Overall, these results from in vitro organ bath tests demonstrated that the hyperplastic prostate strips had weakened force generation and were more characteristic of a tonic contractile phenotype with enhanced force maintenance.

The Inconsistency of the current study with our previous research further highlights the complicated pathogenesis of BPH, which needs to be discussed in detail. Significant pleomorphisms have been histologically observed during different phases of BPH development [25]. The early stage of BPH is characterized by the formation of new glandular and stromal nodules in equal proportions. The impaired balance between the proliferation and apoptosis of the epithelial and mesenchymal cells may contribute to this early phase [32,33]. In established diseases, however, the proportion of the glandular component clearly predominates. Moreover, early studies have demonstrated that transition zone tissues, which contribute to gland size and are the target of surgical resection, exhibited glandular component proliferation and reduced stoma [34]. It has also been well-studied that tissues from a small prostate demonstrate the predominance of fibromuscular stroma, and large glands from enucleation surgeries primarily showed epithelial nodules [32]. The tissues used in our current study were derived from patients with enlarged prostates (>50 mL) undergoing surgeries. Epithelial hyperplasia was predominant in 18 (60.0%) cases, and stromal hyperplasia was predominant in only five (16.7%) patients. This was significantly different from our previous study, in which hyperplastic prostates had obvious stromal hyperplasia with increased SM cells and collagen fibers. We found that differentiated SM cells expressing MHC were rarely distributed in the stroma of the enlarged prostate, which may lead to impaired contraction generation. In the study by Castro et al. [35], prostatic epithelial cells from human BPH tissues showed impaired cell senescence, consistent with the decreased secretory activity of differentiated epithelial cells. It was unclear whether the decreased expression of MHC and contractile function represents the senescence impairment of SM cells [36]. However, we previously indicated that strips from small glands exhibited greater force generation after KCl and PE stimulation. This could explain the clinical phenomenon that for patients in early-stage BPH with small glands, α-adrenoceptor agonists that relax the contraction of SM are remarkably effective, and these are not as effective in patients with large prostates. The abundant expression of NMMHC-B in the epithelial cells of the enlarged prostate may reflect characteristics of proliferation. In fact, a study has revealed that proliferation markers (PCNA and Ki67) could only be detected in glandular contents. Thus, the proliferation of mesenchymal cells can be assumed to originate from an epithelial–mesenchymal transition modulated by TGF-β [37,38]. SM plays a crucial role in the pathophysiology of BPH/LUTS despite the deterioration of the contractile apparatus and force generation in the enlarged prostate. In enlarged glands, the prostatic SM is more characteristic of a tonic type and is accompanied by increased tension maintenance, which may cause constant tension of the prostatic urethra even for baseline levels of neurotransmitter release. In the regression analysis, we found that the tonic contraction subtype SM-A was associated with patients’ Qmax, and SM-1 was correlated with patients’ IPSS. Moreover, the degradation of SM MHC affected patients’ IPSS.

Regulatory networks were built to subsequently explore the regulatory factors of TFs and microRNAs targeting the MYH11, which encodes SM MHC, and MYH10, which encodes NMMHC-B, in the human prostate. Then, 14 DETFs and 15 DEMs were used to build the regulatory networks. Afterward, miR-106a-5p and two TFs (KLF6 and NR4A2) were further analyzed because they may modulate the expression of MHC. We validated the miR-106a-5p-KLF6-MYH11 and miR-106a-5p-NR4A2-MYH11 pathways in vitro using WPMY-1 cell lines. These regulatory factors may provide new therapeutics targeting SMM alterations in prostates of BPH patients in the future, but the mechanism needs to be fully elucidated by further studies. 

One limitation of our study is that the testosterone-induced rat BPH model may not completely replicate the pathological changes seen in human hyperplastic prostates despite the observed increase in rat prostate size and histological alterations. We observed no differences in the composition of SMM isoforms in the rat BPH model, and the expression of MHC and NMMHC isoforms did not align with that of enlarged human prostates. Therefore, a more suitable animal model should be sought in the future. Another significant limitation pertains to the regulatory network. The DETFs and DEMs were identified from gene expression profiling (GSE119195) and a previous study, although the dataset was uploaded by our team. Consequently, inconsistencies may arise due to variations in sample sources. We focused solely on the miR-106a-5p-KLF6-MYH11 and miR-106a-5p-NR4A2-MYH11 pathways to preliminarily validate the regulation of factors targeting MYH11 in WPMY-1 cell lines. To our knowledge, there have been no studies investigating the modulation of miR-106a-5p with KLF6 or NR4A2 on MYH11. Therefore, further research is necessary to confirm the impact of these factors on MYH11 in prostatic SM in vivo.

## 5. Conclusions

In conclusion, our current study supplements our previous report, wherein we claimed that T-induced BPH rats with prostatic epithelial hyperplasia showed upregulation of MHC and NMMHC-A with no changes in SMM isoforms. However, in the enlarged prostates of patients undergoing surgeries, MHC expression was significantly decreased compared to normal tissue, with elevated levels of SM-1, MLC_17b_, and NMMHC-B isoforms. Moreover, the alterations of SMM and NMM may contribute to the tonic contractile characteristic of the prostatic SM and the LUTSs of BPH patients. Lastly, the miR-106a-5p-KLF6-MYH11 and miR-106a-5p-NR4A2-MYH11 pathways in the prostatic SM may be potential regulatory mechanisms and need to be further investigated.

## Figures and Tables

**Table 1:** Primer sequences used to amplify target genes by PCR.

Target Gene		Primer	Primer Sequence
Rat	SM-1/-2	Forward	5′-GCTGGAAGAGGCCGAGGAGGAATC-3′
		Reverse	5′-GAACCATCTGTGTTTTCAATAA-3′
	SM-A/-B	Forward	5′-GGCCTCTTCTGCGTGGTGGTC-3′
		Reverse	5′-TTTGCCGAATCGTGAGGAGTTGTC-3′
	MLC17a/17b	Forward	5′-GAGAGTGGCCAAGAACAA-3′
		Reverse	5′-CAGCCATTCAGCACCATGCG-3′
	MHC	Forward	5′-TTTGCCATTGAGGCCTTAGG-3′
		Reverse	5′-GTTCACACGGCTGAGAATCCA-3′
	NMMHC-A	Forward	5′-CTGAACGACAACATTGCCA-3′
		Reverse	5′-GATTCGATCCACATCCTTCC-3′
	NMMHC-B	Forward	5′-TGAGAAGCCGCCACACATC-3′
		Reverse	5′-CACCCGTGCAAAGAATCGA-3′
	NMMHC-C	Forward	5′-ACTCTGGGCTCATTTATACCT-3′
		Reverse	5′-CCTCCGTGTAGATAGGCAG-3′
	GAPDH	Forward	5′-AACTCCCATTCTTCCACCT-3′
		Reverse	5′-TTGTCATACCAGGAAATGAGC-3′
Human	SM-1/-2	Forward	5′-GCTGGAGGAGGCAGAGGAGGAGTC-3′
		Reverse	5′-GAACCATCTGCATTTTCAATAA-3′
	SM-A/-B	Forward	5′-GGCCTCTTCTGCGTGGTGGTC-3′
		Reverse	5′-TTTGCCGAATCGTGAGGAGTTGTC-3′
	MLC17a/17b	Forward	5′-GAGAGTGGCCAAGAACAA-3′
		Reverse	5′-CAGCCATTCAGCACCATGCG-3′
	MHC	Forward	5′-CAGTTGTTAGCCGAGGAGAAA-3′
		Reverse	5′-GCTTTGAGCATTTTGTTGGTC-3′
	NMMHC-A	Forward	5′-GTGCACTGGTGAATCTGGAG-3′
		Reverse	5′-TGGTCCTTCTTGCTCTTGTG-3′
	NMMHC-B	Forward	5′-TCTTGGGATGAATGTGATGG-3′
		Reverse	5′-TATTGATGCGATGAACGAGC-3′
	NMMHC-C	Forward	5′-CAGCCGTCAAATGCAAACC-3′
		Reverse	5′-ACCCTCCTCAGCCTCAAGGT-3′
	GAPDH	Forward	5′-TCAAGATCATCAGCAATGCC-3′
		Reverse	5′-CGATACCAAAGTTGTCATGGA-3′

**Table 2:** List of primary antibodies.

Target Protein	Name of Antibody	Manufacturer, Catalog	Species Raised in: Monoclonal or Polyclonal	Reactivity	Dilution Used
MHC	MYH11 antibody	Santa Cruz, sc-6956	Mouse monoclonal	Mouse, rat, human	1:1000 (WB), 1:100 (IF)
NMMHC-A	Anti-non-muscle Myosin IIA antibody	Abcam, ab75590	Rabbit polyclonal	Mouse, Rat, Human	1:1000 (WB), 1:100 (IF)
NMMHC-B	Anti-non-muscle Myosin IIB antibody	Abcam, ab230823	Rabbit monoclonal	Mouse, Rat, Human	1:1000 (WB), 1:100 (IF)
NMMHC-C	Anti-Myh14 antibody	Abcam, ab232897	Rabbit polyclonal	Rat, Human	1:1000 (WB), 1:100 (IF)
KLF6	Anti-KLF6 antibody	Abcam, ab241385	Rabbit polyclonal	Human	1:1000 (WB), 1:100 (IF)
NR4A2	Nurr1 antibody (447C2a)	Santa Cruz, sc-81345	Mouse monoclonal	Human	1:1000 (WB), 1:100 (IF)
GAPDH	GAPDH antibody (G-9)	Santa Cruz, sc-365062	Mouse monoclonal	Mouse, Rat, Human	1:1000 (WB)

**Table 3:** Variation in biometric and physiological parameters in control and BPH rats.

Group	Body Weight (g)	Ventral Prostate Weight (mg)	Seminal Vesicles Weight (mg)	Prostate Index	T Level (ng/mL)
Control	429.8 (32.7)	778.3 (135.3)	1001.8 (288.5)	1.8 (0.3)	4.4 (0.4)
BPH	387.2 (13.7) **	1231.3 (131.4) **	2483.9 (360.6) **	3.2 (0.3) **	6.2 (0.5) *

*p*-values were calculated using unpaired *t* tests. Data were mean ± SD. ** *p* < 0.01 vs. control, * *p* < 0.05 vs. control.

**Table 4:** Demographics and preoperative parameters of the 30 included patients.

Mean *yrs* age (range)	71.2 (51–84)
Mean *mL* PV (range)	61.5 (50.5–105.4)
No. pathology (%)	
epithelial hyperplasia	18 (60.0)
Stromal hyperplasia	5 (16.7)
epithelial-stromal hyperplasia	7 (23.3)
No. hypertension (%)	6 (20.0)
No. diabetes (%)	4 (13.3)
No. surgery type (%)	
open	5 (16.7)
TURP	16 (53.3)
TUVP	9 (30.0)
No. AUR (%)	7 (23.3)
Mean *mL* PVR (range)	117.5 (0–548)
Mean *mL*/*s* Qmax (range)	7.4 (2.2–13.5)
Mean IPSS (range)	22.1 (12–30)

**Table 5:** Results of regression models for evaluating the effect of the factors on PVR, AUR, Qmax, and IPSS.

Factors	PVR		AUR		Qmax		IPSS	
	Standardized Coefficient	Adjusted by Age, PV, Pathology, Hypertension, and Diabetes	Odds Ratio	Adjusted by Age, PV, Pathology, Hypertension, and Diabetes	Standardized Coefficient	Adjusted by Age, PV, Pathology, Hypertension, and Diabetes	Standardized Coefficient	Adjusted by Age, PV, Pathology, Hypertension, and Diabetes
Age	−0.538 *	-	1.190	-	0.041	-	0.612 **	-
PV	0.589 *	-	1.034	-	−0.036	-	0.075	-
Patho EpithelialStromal	−0.076	-	1.923	-	−0.289	-	−0.236	-
	0.365	-	0.792	-	0.364	-	0.118	-
Hypertension	−0.256	-	1.900	-	−0.175	-	0.189	-
Diabetes	−0.154	-	4.200	-	0.364	-	0.287	-
SM-1 percent	−0.047	0.152	9.812	132.426	−0.107	−0.232	0.624 **	0.628 ***
SM-A percent	0.309	0.379 *	0.008	17.295	−0.641 *	−0.734 *	0.050	0.135
MLC_17b_ percent	0.197	0.037	65.347	577.897	−0.381	−0.368	0.080	0.209
MHC	−0.353	−0.510	0.034	0.016	−0.217	−0.434	−0.439 *	−0.604 **
NMMHC-A	0.240	0.082	0.292	0.325	0.239	−0.010	−0.109	0.310
NMMHC-B	0.580 *	0.563	0.106	0.106	−0.113	−0.084	0.598 **	0.446
NMMHC-C	0.580 *	0.542	0.259	0.350	0.277	0.202	−0.384	−0.168

Linear regression models were employed to evaluate the influencing factors on PVR, Qmax, and IPSS. When the dependent variable was AUR, the logistic regression model was used. Significant difference was showed as boldface. * *p* < 0.05; ** *p* < 0.01.*** *p* < 0.001 vs. control

## Data Availability

The data used to support the findings of this study are available from the corresponding author upon request.
